# Vitamin D Protects against Oxidative Stress and Inflammation in Human Retinal Cells

**DOI:** 10.3390/antiox9090838

**Published:** 2020-09-08

**Authors:** Patricia Fernandez-Robredo, Jorge González-Zamora, Sergio Recalde, Valentina Bilbao-Malavé, Jaione Bezunartea, Maria Hernandez, Alfredo Garcia-Layana

**Affiliations:** 1 Retinal Pathologies and New Therapies Group, Experimental Ophthalmology Laboratory, Department of Ophthalmology, Clinica Universidad de Navarra, 31008 Pamplona, Spain; pfrobredo@unav.es (P.F.-R.); jgzamora@unav.es (J.G.-Z.); vbilbao@unav.es; (V.B.-M.)jbezunartea@unav.es (J.B.); mahersan@unav.es (M.H.); aglayana@unav.es (A.G.-L.); 2 Navarra Institute for Health Research, IdiSNA, 31008 Pamplona, Spain; 3 Red Temática de Investigación Cooperativa Sanitaria en Enfermedades Oculares (Oftared), 31008 Pamplona, Spain

**Keywords:** vitamin D, oxidative stress, inflammation, diabetic retinopathy

## Abstract

Diabetic retinopathy is a vision-threatening microvascular complication of diabetes and is one of the leading causes of blindness. Oxidative stress and inflammation play a major role in its pathogenesis, and new therapies counteracting these contributors could be of great interest. In the current study, we investigated the role of vitamin D against oxidative stress and inflammation in human retinal pigment epithelium (RPE) and human retinal endothelial cell lines. We demonstrate that vitamin D effectively counteracts the oxidative stress induced by hydrogen peroxide (H_2_O_2_). In addition, the increased levels of proinflammatory proteins such as Interleukin (IL)-6, IL-8, Monocyte chemoattractant protein (MCP)-1, Interferon (IFN)-γ, and tumor necrosis factor (TNF)-α triggered by lipopolysaccharide (LPS) exposure were significantly decreased by vitamin D addition. Interestingly, the increased IL-18 only decreased by vitamin D addition in endothelial cells but not in RPE cells, suggesting a main antiangiogenic role under inflammatory conditions. Moreover, H_2_O_2_ and LPS induced the alteration and morphological damage of tight junctions in adult retinal pigment epithelium (ARPE-19) cells that were restored under oxidative and inflammatory conditions by the addition of vitamin D to the media. In conclusion, our data suggest that vitamin D could protect the retina by enhancing antioxidant defense and through exhibiting anti-inflammatory properties.

## 1. Introduction

Diabetic retinopathy (DR) is the main cause of blindness among adults of working age globally [1]. An effective management of diabetes reduces the risk of complications, however, poor control of the condition can result in microvascular complications [2]. Nevertheless, even patients with intensive glycemic control have rate of progression over 7% [3]. The Diabetes Control and Complications Trial (DCCT) found that intensive glycemic control can effectively reduce or slow down the development or progression of DR by 76% in patients with type 1 diabetes, while the U.K. Prospective Diabetes Study (UKPDS) came to a similar conclusion in patients with type 2 diabetes [2,4].

The investigation of the underlying mechanisms of DR is of great importance and may provide potential new alternative treatments. Several studies have shown that diabetes can lead to DR by several mechanisms including the polyol pathway [5], non-enzymatic glycation [6], activation of protein kinase C [7], oxidative stress [8,9,10,11,12], and inflammation [13]. Oxidative stress is considered to be one of the crucial causative factors in the development of DR, in combination with other biochemical imbalances, leading to both structural and functional changes and also promoting an increased loss of capillary cells in the microvasculature of the retina [11,12]. In addition, a significant body of evidence supports the role of proinflammatory cytokines, chemokines, and other inflammatory mediators in the pathogenesis of DR, leading to chronic low-grade inflammation of the retina and eventually to neovascularization [14].

Vitamin D (VITD) is a fat-soluble molecule that is found in two forms: vitamin D_2_ and vitamin D_3_ [15]. Vitamin D_3_ is well recognized as a secosteroid hormone that regulates many cellular signaling activities through its nuclear VITD receptor (VDR) in target cells [15]. Previous studies reported that VITD treatment can protect cells and tissues from oxidative damage [16] and it has also been found to prevent oxidative stress damage in DR in a high-glucose environment [17,18] and in diabetic rats [19]. In addition, some polymorphisms in the VITD receptor gene are associated with increased risk of DR [20,21,22,23] and VITD-deficient patients have increased risk of DR [24,25]. VITD also appears to be beneficial in other retinopathies such as age-related macular degeneration (AMD) [26,27,28]. Therefore, VITD may be suggested as a useful candidate for diabetic patients to reduce the pathological complications of diabetes. However, further research needs to be made to clarify the possible therapeutic potential of VITD in the DR.

In the current study, we investigated the safety of VITD in adult retinal pigment epithelium (ARPE-19) and human retinal endothelial (HREC) cell lines, focusing on its antioxidant and anti-inflammatory effect on cell integrity, oxidative stress, and cytokines release. This study constitutes an in vitro evaluation of the molecular pathways by which VITD might tackle the oxidative stress and inflammation observed in patients suffering from retinal pathologies such as DR.

## 2. Materials and Methods

### 2.1. Expression of Genes Related to VITD Metabolism

Total RNA was isolated from cell lines using an ABI PRISM 6100 Nucleic Acid PrepStation (Life Technologies, Carlsbad, CA, USA). Subsequently, the quantity and quality of purified messenger RNA (mRNA) was checked using a NanoDrop spectrophotometer (Nanodrop Technologies, Montchanin, DE, USA) at 260/280. Using the qScript cDNA Supermix Kit (Quanta Biosciences, Inc., Gaithersburg, MD, USA), we reverse-transcribed 1000 ng of each mRNA under the manufactured conditions. The primers of the relevant genes are as follows: cytochrome P450 *(CYP)27A1* (Unigene ID-Hs.516700, 5′-GGCAAGTACCCAGTACGG-3′ and 5′-AGCAAATAGCTTCCAAGG-3′), *CYP27B1* (Unigene ID-Hs.524528, 5′-CACCTGACCCACTTCCTGTT-3′ and 5′-TCTGGGACACGAGAATTTCC-3′), *CYP2R1* (Unigene ID-Hs.371427, 5′-AGAGACCCAGAAGTGTTCCAT-3′ and 5′-GTCTTTCAGCACAGATGAGGTA-3′), *CYP24A1* (Unigene ID-Hs.89663, 5′-CCCACTAGCCACCTCGTACCAAC-3′ and 5′-CGTAGCCCTTCTTTGCGGTAGTC-3′), *VDR* (Unigene ID-Hs.524368, 5′-CGCTCCAATGAGTCCTTCACC-3′and 5′-GCTTCATGCTGCACTCAGGC-3′), *Cubilin* (Unigene ID-Hs.166206, 5′-GCGGCTTCACTGCTTCCTA-3′ and 5′-GAGTGATGGTGTGCCCTTGT-3′), *Megalin* (Unigene ID-Hs.657729, 5′-TAAGTCAGTGCCCAACCTTT-3′and 5′-GCGGTTGTTCCTGGAG-3′). A 2720 Thermal Cycler (Life Technologies, Gaithersburg, MD, USA) was used for amplification with the following protocol: 10 min at 95 °C, 40 cycles of 30 s at 95 °C, 1 min 58 °C, and extension of 45 s at 72 °C. Two housekeeping genes, *18S* (Unigene ID-Hs.99999901_s1, and glyceraldehyde 3-phosphate dehydrogenase, *GAPDH* (Unigene ID-Hs.99999905_m1), Life Technologies, Gaithersburg, MD, USA) were used as internal controls, and 18S (5′-GTTGGTGGAGCGATTTGTCT-3′ and 5′-GGCCTCACTAAACCATCCAA-3′) was selected as the best control.

### 2.2. Cell Culture

Human retinal pigment epithelial cells, ARPE-19 (CRL-2302, ATCC, Manassas, VA, USA), and human retinal endothelial cells, HREC (p10880, Innoprot, Vizcaya, Spain), were used. ARPE-19 cells (three passages) were grown to confluence (37 °C, 5% CO_2_) in Dulbecco’s modified Eagle’s medium (DMEM; D6429, Sigma-Aldrich, St. Louis, MO, USA) containing 10% fetal bovine serum (FBS; 10270106 Gibco ThermoFisher, Paisley, UK), 1% fungizone (Gibco, Carlsbad, CA, USA), and penicillin–streptomycin (Gibco, Carlsbad, CA, USA). HREC cells were seeded in T75 flasks (353136, Falcon, Corning Life Science, Tewksbury, MA, USA) covered with 1 mg/mL of fibronectin (Innoprot, p8248, Vizcaya, Spain) and grown to confluence in a standard incubator at 37 °C under humidified 5% CO_2_ conditions in Endothelial Cell Medium (Innoprot, p60104, Vizcaya, Spain) containing 5% FBS (Innoprot, Vizcaya, Spain), 1% Endothelial Cell Grow Supplement (ECGS, Innoprot, Vizcaya, Spain), and penicillin–streptomycin solution (Innoprot, Vizcaya, Spain).

### 2.3. Validation of the Cell Lines: Stable Phenotypic Characterization

To verify that ARPE-19 and HREC cells preserved their phenotype, we performed retinoid isomerohydrolase *RPE65* (RPE65) (1:100, 78036, Abcam, Cambridge, MA, USA) and caveolin (1:250, 3238S, Cell Signaling, Danvers, MA, USA) staining by immunofluorescence. Briefly, 100,000 ARPE-19 and 50,000 HREC cells were seeded on a 10 mm dish (Menzel-Glaser, Waltham, MA, USA). Cold methanol was used for cellular fixing. Afterward, cells were washed with 1% phosphate buffer saline (PBS) and then incubated with blocking buffer containing 1% bovine serum albumin (BSA), 0.5% Triton X-100, 0.2% sodium azide, and 1% fetal bovine serum (FBS) for 1 h at 4 °C. Cells were incubated with the primary antibodies, diluted in blocking buffer at 4 °C for 24 h, and washed once more with PBS and then incubated with the secondary fluorescent antibodies goat anti-mouse 488 (1:250, A11029, Life technologies, Gaithersburg, MD, USA) and donkey anti-rabbit 488 (1:250, A21206, Invitrogen, Carlsbad, CA, USA) for RPE65 marker diluted in blocking buffer during 1 h in the dark. Nuclei were labelled with 4′,6-diamidino-2-phenylindole (DAPI; Sigma-Aldrich, St. Louis, MO, USA). The morphology of cells was observed under an inverted phase-contrast microscope (Olympus CKX41, Tokyo, Japan) and photographed by a digital camera, and fluorescent images were obtained using a confocal microscope (LSM800, Zeiss, Oberkochen, Germany).

### 2.4. Treatments and Experimental Design: Oxidative Stress and Inflammation-Like Conditions

ARPE-19 and HREC cell lines were treated with VITD (1 nM; C9756-1G, Sigma-Aldrich, St. Louis, MO, USA) for 1 h to test its effect on cells. To induce in vitro oxidative stress, we subjected cells to H_2_O_2_ (1000 µM, Panreac, Barcelona, Spain) for 2 h. To evaluate the protective effect of VITD, we added it (1 nM; Sigma-Aldrich, St. Louis, MO, USA) in concomitance 1 h before the end of the induction time. Lipopolysaccharide (LPS; Sigma-Aldrich, St. Louis, MO, USA) was added for 24 h (20 µg/mL for ARPE-19 and 50 µg/mL for HREC cells) to induce an inflammatory response, and then VITD (1 nM; Sigma-Aldrich, St. Louis, MO, USA) was added to the supernatant for 1 h in concomitance.

### 2.5. Cell Structure and Integrity: Zonula Occludens (ZO)-1 Immunofluorescence and Western Blot

The effect of VITD on intercellular tight junction status was evaluated by zonula occludens-1 (ZO-1) immunofluorescence. One-hundred thousand ARPE-19 cells per well were seeded on laminin-coated polycarbonate membrane cell culture inserts (Corning Life Science, Tewksbury, MA, USA) and were grown in 1% FBS-DMEM for 4 weeks. Immunofluorescence was then performed using a ZO-1 anti-rabbit Alexa Fluor 594 antibody (1:100, 339194, Invitrogen-Life Technologies, Gaithersburg, MD, USA) diluted in blocking buffer, following the same protocol described above. DAPI (4′,6-diamidino-2-phenylindole; Sigma-Aldrich, St. Louis, MO, USA) was used to stain cell nuclei. Images were obtained with a laser scanning confocal imaging system (LSM800, Zeiss, Oberkochen, Germany). H_2_O_2_ (1600 µM, Panreac, Barcelona, Spain) over 6 h was used as a positive control for oxidative stress conditions, and LPS (20 µg/mL; L2880 Sigma-Aldrich, St. Louis, MO, USA) was used for 24 h for inflammatory conditions.

A total of 5 µg of ARPE-19 cell homogenates from three passages were mixed with NuPage (4x, Bio-Rad, Hercules, CA, USA), boiled for 5  min, separated on 7% sodium dodecyl sulfate polyacrylamide gel electrophoresis (SDS-PAGE) gels, and transferred onto a nitrocellulose membrane. After we blocked them with 5% skimmed milk (*w*/*v*; Scharlau, Barcelona, Spain), 0.1% Tween-20 (*w*/*v*; Sigma-Aldrich, St. Louis, MO, USA) in tris buffer saline (TBS) for 1 h at room temperature (RT), membranes were exposed to the mouse monoclonal ZO-1 antibody (1:1000, #33-9100, Invitrogen, Carlsbad, CA, USA) at RT for 1 h, followed by a horseradish peroxidase-conjugated goat anti-mouse antibody (sc-2005; 1:5000, 1 h, RT Santa Cruz Biotechnology Inc., Santa Cruz, CA, USA). Signal was detected with an enhanced chemoluminescence (ECL) kit (ECL-Select, #RPN2235, GE Healthcare, Fairfield, CT, USA) and images were captured with ImageQuant 400 (GE Healthcare). The relative intensities of the immunoreactive bands were analyzed with ImageQuant TL (GE Healthcare, Fairfield, CT, USA). The loading was verified by Ponceau S red and an anti-β-actin monoclonal antibody (1:10,000, 1 h, RT; Sigma-Aldrich, St. Louis, MO, USA), followed by a goat anti-mouse antibody (sc-2005; 1:10,000, 5% skimmed milk, 1 h, RT; Santa Cruz Biotechnology Inc., Santa Cruz, CA), and signal was detected using ECL-Prime, #RPN2232 (GE Healthcare, Fairfield, CT, USA). Data are presented as absorbance units (AU) ZO-1/β-actin (% vs. saline).

### 2.6. Assay to Detect Cell Apoptosis

Apoptosis in ARPE-19 and HREC was performed in cultured plates using an in situ cell death detection kit with TMR Red according to the manufacturer’s instructions (#12156792910, Roche, West Sussex, UK) and stored at 4 °C until analysis, with the apoptotic cells being labelled with active caspase-3 antibody (1:100, G7481; Promega, Madison, Wisconsin, USA) using the protocol mentioned above and incubated with the secondary fluorescent antibody donkey anti-rabbit 488 (A21206, Invitrogen). Nuclei were labelled with DAPI and images were obtained using a confocal microscope (LSM800, Zeiss, Oberkochen, Germany). H_2_O_2_ (600 µM, Panreac, Barcelona, Spain) for 2 h was used as positive control for oxidative stress conditions.

### 2.7. Viability/Toxicity Assay (MTT)

Cell viability/toxicity in ARPE-19 and HREC cell lines was determined by the 3-(4,5-dimethylthiazol-2-yl)-2,5-diphenyltetrazolium bromide (MTT) reduction assay CellTiter 96 AQueous One Solution Cell Proliferation Assay (Promega, Madison, WI, USA), following the manufacturer’s instructions. A total of 10,000 ARPE-19 or HREC cells were grown until confluence in DMEM with 10% FBS onto 96-well plates. Then, cells were cultivated for 1 additional week in serum-reduced medium (1% FBS-DMEM), and VITD was added to the culture medium for 24 h at 1, 5, 10, and 50 nM doses.

### 2.8. Proliferation Assay (Bromodeoxyuridine, BrdU)

To examine the effect of VITD on ARPE-19 and HREC cell proliferation, we seeded 10,000 cells onto 96-well plates. After 24 h, cells were exposed to VITD (1 nM) for 1 h and the Calbiochem BrdU Cell Proliferation Assay (Calbiochem, La Jolla, CA, USA) was performed in accordance with the manufacturer’s instructions.

### 2.9. Measurement of 8-Hydroxidioxiguanosine (8-OHdG) under Oxidative Stress Conditions

Oxidative damage was measured in ARPE-19 and HREC supernatants subjected to oxidative stress conditions, as described above. To evaluate the effect of VITD, we added 1 nM to the media. Supernatants (100 µL) were evaluated by using the Enzyme-Linked ImmunoSorbent Assay (ELISA) kit #ab201734 (Abcam, Cambridge, MA, USA). Data are presented in nanograms per milliliter (ng/mL).

### 2.10. Multiplex Cytokine Analysis under Inflammatory and Basal Conditions: Interleukin (IL)-1β, IL-6, IL-8, IL-10, IL-12p70, and IL-18; Interferon (IFN)-γ; Monocyte Chemoattractant Protein (MCP)-1; and Tumor Necrosis Factor (TNF)-α

The cytokine analysis for IL-1β, -6, -8, -10, -12p70, and -18; IFN-γ; MCP1; and TNF-α was made using FirePlex Firefly (Abcam, Cambridge, MA, USA) particle multiplex immunoassay for Flow Cytometry and Analysis Workbench, a software for multiplex protein expression assays from Abcam Laboratories. Supernatants were used for this purpose and were measured under inflammatory conditions as abovementioned. All cytokines are expressed in pictograms per milliliter (pg/mL), with the exception of MCP-1 and IL-8, which were expressed in ng/mL.

### 2.11. Statistical Analysis

All parameters were subjected to analysis of the variance (ANOVA) test followed by the Bonferroni post-hoc for multiple comparisons. A difference *p* < 0.05 was considered statistically significant. GraphPad Prism 6.0 (GraphPad Prism Software Inc., San Diego, CA, USA) was used for statistical analysis.

## 3. Results

### 3.1. Confirmation of VITD Receptor Expression in ARPE-19 and HREC Cell Lines

In this study, we demonstrated that ARPE-19 and HREC cell lines expressed the machinery for vitamin D_3_ and could produce 1,25(OH)_2_D_3_. This is the first time that the expression of Vit D_3_-synthesizing components has been reported in HREC cells.

**Figure 1:**
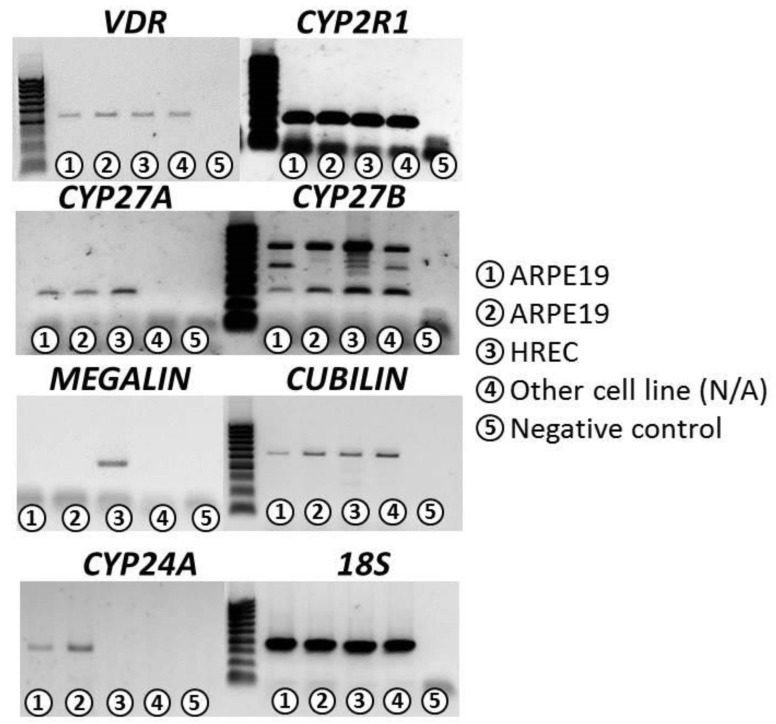
Conventional PCR was carried out using total RNA extracted from ARPE-19 (lines 1 and 2) and HREC cells (line 3). We analyzed the gene expression of *VDR*, *CYP27B1*, *CYP24A1*, *CYP27A1*, *CYP2R1*, *cubilin*, and *megalin*. Ribosomal 18S was used as an internal PCR control. Results are representative of at least three independent experiments. Molecular sizes (base pairs [bp]): *18S* (400), *VDR* (421), *CYP27B1* (302), *CYP24A1* (485), *CYP27A1* (292), *CYP2R1* (259), *cubilin* (518), and *megalin* (290).

We performed conventional polymerase chain reaction (PCR) experiments in order to determine the expression of the following genes involved in VITD synthesis. ARPE-19 cell line highly expressed the genes cytochrome P450 *(CYP)2R1*, *CYP27B*, and *CYP24A* and showed a low expression of vitamin D receptor (*VDR*), *CYP27A*, and *cubilin* genes; however, they did not express the *megalin* gene. HREC cell line highly expressed the genes *CYP2R1* and *CYP27B*. HREC showed a lower expression of *VDR*, *CYP27A*, *cubilin*, and *megalin* genes and did not express the *CYP24A* gene (Table 1). Ribosomal 18S was used as an internal PCR control (Figure 1).

### 3.2. Validation of the Cell Lines: Stable Phenotypic Characterization

**Figure 2:**
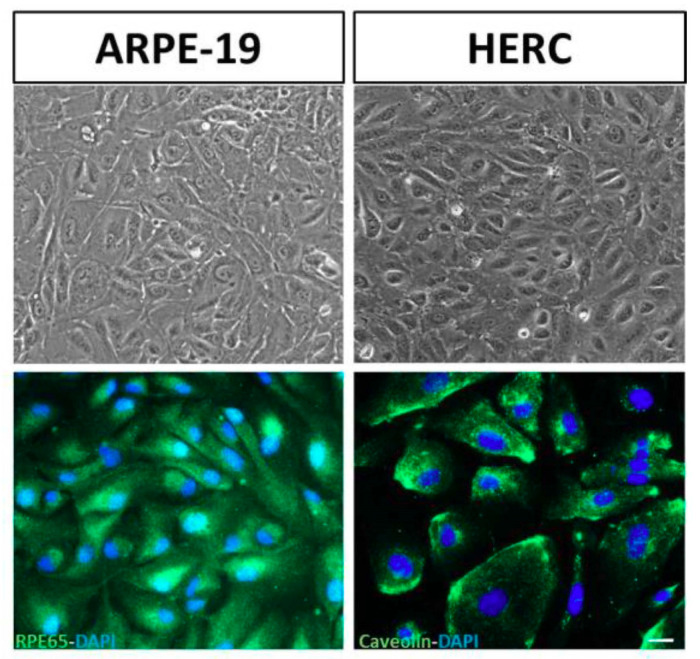
ARPE-19 and HREC cells’ phenotyping. Immunofluorescence of RPE65 (green) and caveolin (green) for ARPE-19 and HREC cell lines’ labelling, respectively. Upper panel shows cells at bright-field microscopy and down panel shows cells under fluorescence microscopy. Nuclei were labeled with 4′,6-diamidino-2-phenylindole (DAPI) (blue). Scale bar: 20 µm.

We performed immunofluorescence for specific ARPE-19 and HREC cell lines’ markers. We used RPE65 protein (Abcam 78036) for ARPE-19 cells and caveolin protein (Cell Signalling 3238S) for HREC cells. No changes in the phenotypic characteristics were found, and the cells expressed all the selected markers (Figure 2).

### 3.3. Effect of VITD Addition on Cytoxicity and Proliferation

**Figure 3:**
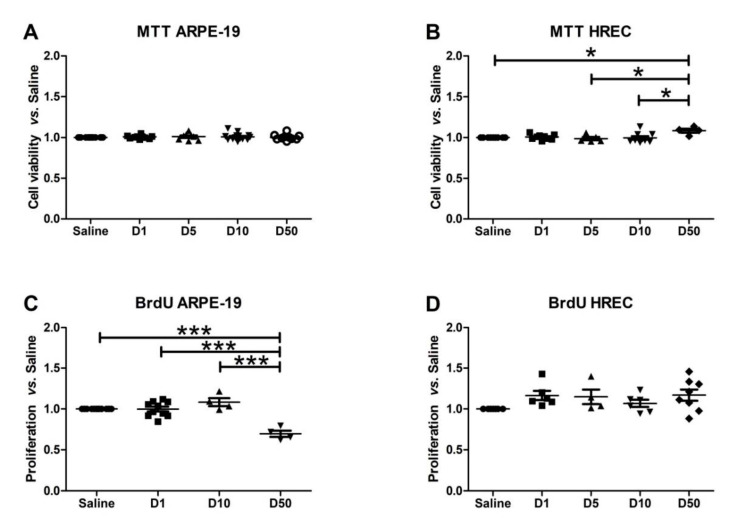
Graphs showing 3-(4,5-dimethylthiazol-2-yl)-2,5-diphenyltetrazolium bromide (MTT) (**A**,**B**) and Bromodeoxyuridine (BrdU) (**C**,**D**) results for ARPE-19 (**A**,**C**) and HREC (**B**,**D**) cells treated with different VITD concentrations (1, 5, 10, and 50 nM) for 1 h. Any VITD dose showed cytotoxic effects on ARPE-19 cells compared to saline group. HREC showed significant alterations in MTT D50 compared to the rest of the groups (* *p* < 0.05). ARPE-19 proliferation was statistically reduced (*** *p* < 0.001) in the highest dose analyzed (50 nM) compared to saline and to all the remaining doses. HREC cells’ proliferation was not affected by any VITD dose.

VITD at 1 nM and for 1 h did not show cytotoxic effects in ARPE-19 cells and HREC cells (Figure 3A,B). Moreover, proliferation was not affected by VITD addition at 1 nM and 1 h of exposure time (Figure 3C,D).

VITD doses ranged from 1 to 50 nM for both cell types tested. All doses were safe for ARPE-19 cells at any time measured, and the highest dose used significantly reduced proliferation (*p* < 0.001). HREC cells showed an increase in viability, with the highest vitamin D dose (*p* < 0.05) response being in the highest dose at 50 nM. In addition, proliferation was reduced in ARPE-19 cells subjected to a 50 nM VITD dose. To be sure that the effects observed in the subsequent analysis were caused by vitamin D itself and that they were not masked by deleterious effects on cell viability and proliferation, we decided to use 1 nM of VITD, which did not affect proliferation and viability.

### 3.4. Effect of VITD Addition on Integrity and Apoptosis of Cells

**Figure 4:**
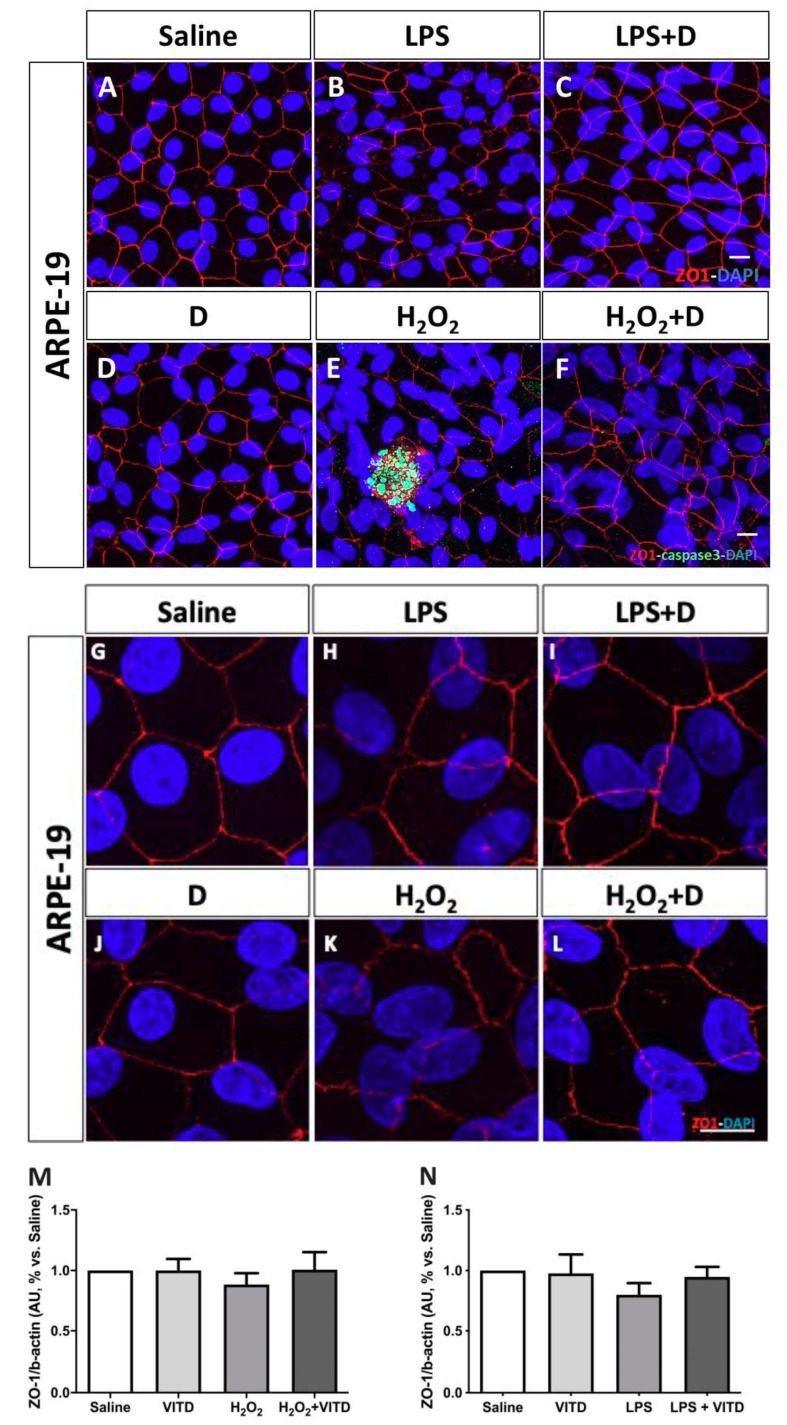
Integrity of ARPE-19 cells evaluated by zonula occludens-1 (ZO-1) (red) and caspase-3 (green) immunofluorescence. VITD (1 nM, 1 h; (**D**)) did not affect ZO-1 structure compared to saline (**A**,**G**). Lipopolysaccharide (LPS) (**B**,**H**) and H_2_O_2_ (**E**,**K**) addition damaged tight junctions and concomitant incubation with VITD (1 nM, 1 h; (**C**,**I**) and (**F**,**L**)) restored the altered structure. (**G–L**) show the apical junction in higher magnification. Caspase-3 was highly observed in the H_2_O_2_ group (**E**) compared to saline (**A**) and VITD (**D**). VITD addition showed restoration, and caspase-3 activation was absent (**F**). Nuclei were labeled with DAPI (blue). Scale bar: 20 µm. Densitometry of ZO-1 expression in ARPE-19 cells under oxidative stress (**M**) and inflammatory (**N**) conditions. Although a tendency to reduce the ZO-1 expression was observed, no statistical differences were found. VITD restored values similar to saline group. *n* = 3.

ARPE-19 cells’ integrity was conserved after adding VITD at 1 nM. Hydrogen peroxide and LPS induced an increase in tortuosity of the junction contacts in ARPE-19 cells that were stabilized in oxidative and inflammatory conditions by the addition of VITD to the media (Figure 4).

LPS and H_2_O_2_ addition showed a tendency to reduce ZO-1 expression, although that difference was not significant. Moreover, the supplementation with VITD partly increased the ZO-1 expression, but this result did not reach statistical significance.

**Figure 5:**
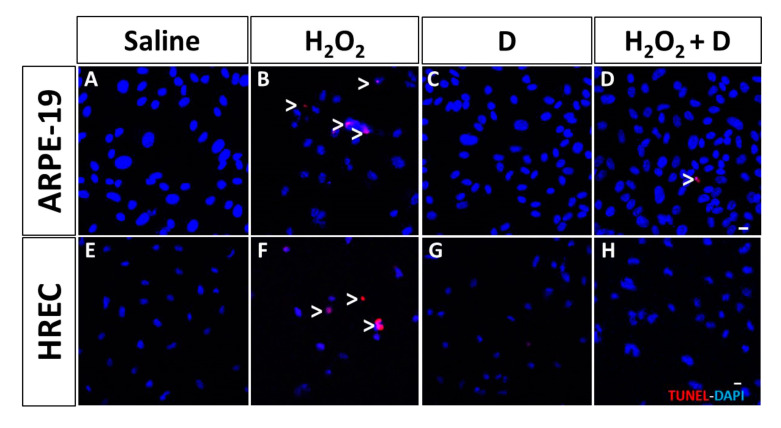
Late apoptosis measured in ARPE-19 (**A**–**D**) and HREC (**E**–**H**) cells by TDT- mediated dUTP-biotin nick end-labeling (TUNEL) and analyzed by fluorescence. TUNEL-positive ARPE-19 and HREC cells were observed after H_2_O_2_ stimulation (2 h; (**B**,**F**)) compared to saline (**A**,**E**). VITD (1 nM, 1 h; (**C**,**G**)) showed similar results to saline groups for both cell types. VITD, in concomitance with H_2_O_2_ (1 h; (**D**,**H**)), showed a reduction in altered nuclei, especially in ARPE-19 cells (**D**), and absence of TUNEL labeling. Nuclei were labeled with DAPI (blue). Scale bar: 20 µm.

Early (caspase-3, Figure 4) and late (TDT- mediated dUTP-biotin nick end-labeling, TUNEL, Figure 5) apoptosis markers revealed that VITD (1 nM) addition did not affect cell death processes. After inflammatory and oxidative induction, ARPE-19 cells and HREC cells showed alterations and an increase labelling for both markers. VITD (1 nM) addition was able to restore those alterations (Figure 5).

### 3.5. Antioxidative and Anti-Inflammatory Properties of VITD Addition

**Figure 6:**
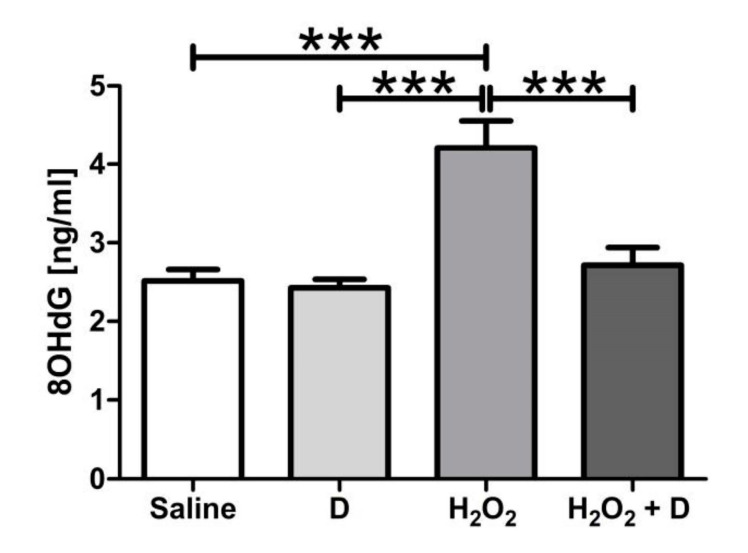
Graph showing 8-OHdG results for ARPE-19 cells measured by ELISA. VITD was able to significantly reduce the 8-OHdG levels elevated by H_2_O_2_ (*** *p* < 0.001). *n* = 3.

Figure 6 shows that oxidative stress induction by H_2_O_2_ significantly (*p* < 0.001) increased 8-OHdG in supernatants from ARPE-19 cells. VITD alone did not modify oxidative damage compared to saline. VITD was able to significantly (*p* < 0.001) reduce 8-OHdG production under oxidative-induced conditions.

**Figure 7:**
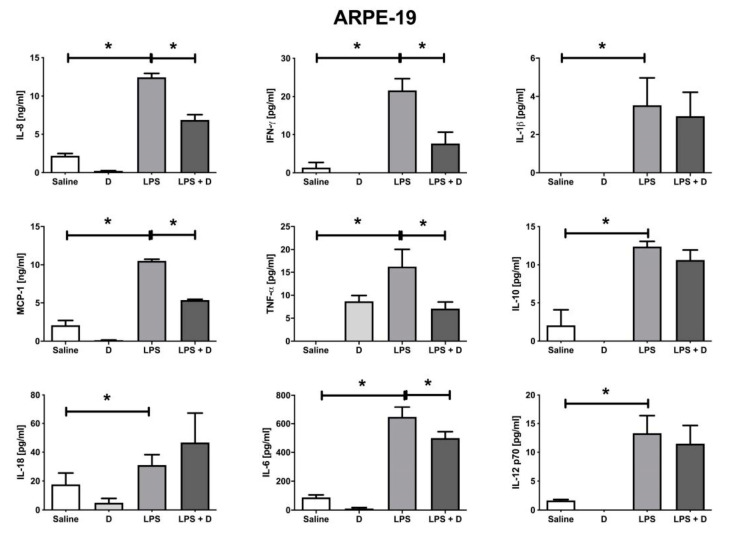
Multiplex inflammatory cytokine array in ARPE-19 cells. All inflammatory cytokine levels were increased by adding LPS (* *p* < 0.05), and Interleukin (IL)-8, Interferon (IFN)-γ, Monocyte chemoattractant protein (MCP)-1, Tumor necrosis factor (TNF)-α, and IL-6 levels were reduced (* *p* < 0.05) with the addition of VITD in concomitance. *n* = 3.

Figure 7 shows that LPS induction increased IL-8, IFN-γ, IL-1β, MCP-1, TNF-α, IL-10, IL-18, IL-6, and IL-12p70 in ARPE-19 cells. Under inflammatory conditions, VITD was able to significantly (*p* < 0.05) reduce IL-8, IFN-γ, MCP-1, TNF-α, and IL-6.

**Figure 8:**
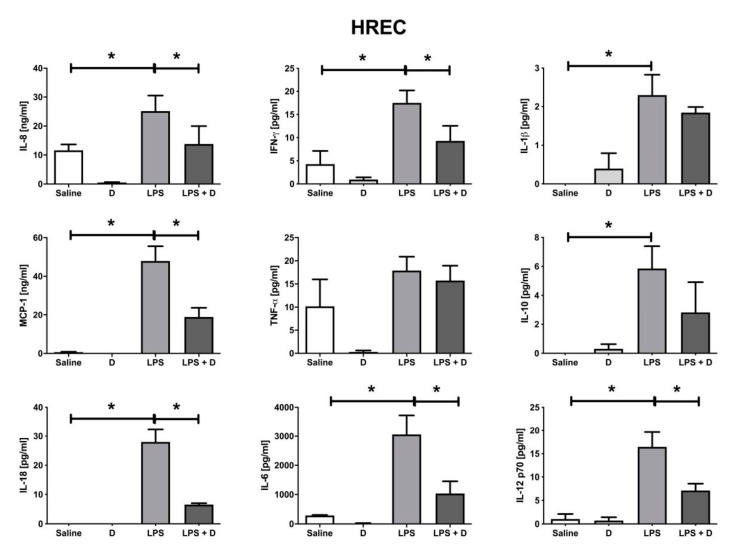
Multiplex inflammatory cytokine array in HREC cells. IL-8, IFN-γ, IL-1β, MCP-1, IL-10, IL-6, and IL-12p70 inflammatory cytokine levels were increased by adding LPS (* *p* < 0.05), and IL-8, IFN-γ, IL-1β, MCP-1, TNF-α, IL-6, and IL-12p70 levels were reduced (* *p* < 0.05) with the addition of VITD in concomitance. *n* = 3.

HREC cells subjected to LPS induction showed an increase in IL-8, IFN-γ, IL-1β, MCP-1, IL-10, IL-6, and IL-12p70 cytokines. VITD significantly (*p* < 0.05) decreased IL-8, IFN-γ, IL-1β, MCP-1, TNF-α, IL-6, and IL-12p70 under inflammatory conditions in HREC cells (Figure 8).

## 4. Discussion

The present study shows that damage observed in human retinal pigmented epithelium (RPE) and retinal endothelium cells under oxidative and inflammatory conditions were restored by the addition of VITD to the media. More specifically, induced inflammatory cytokine levels, early and late apoptosis, and oxidative stress markers were reduced back to control levels. This result suggests that VITD could be a useful candidate in modulating the chronic low-grade inflammation and oxidative stress responsible for the complications in retinal pathologies involving RPE and endothelial cells.

It is well established that glycemic control is an effective management to lower the incidence of complications such as DR. However, even an intensive glycemic control is not sufficient to prevent diabetic microvascular pathologies in all patients [4], and hyperglycemia on its own is not sufficient to trigger widespread diabetic microvascular pathologies in all patients [29,30,31]. Diabetic patients with an initial poor glycemic control have persistent higher incidence of diabetic complications after glucose normalization, a phenomenon described as metabolic memory, suggesting that oxidative stress, non-enzymatic glycation of proteins, epigenetic changes, and chronic inflammation may play a major role in the development and progression of diabetic microvascular complications [32,33] such as diabetic kidney disease [34,35,36], diabetic polyneuropathy [37,38], and DR [9,10,11,14]. VITD deficiency is related to a higher risk of DR in type 1 and 2 diabetes mellitus [24,39]. Apart from its role in tissues related to calcium homeostasis [40], high levels of VDR are also present in inflammatory cells such as dendritic cells, macrophages, T-cells, and B-cells, thus supporting the fact that VITD may have a role in inflammatory and immune responses [41].

25-Hydroxylase (encoded by the *CYP27A1* and *CYP2R1* genes), a cytochrome P450 enzyme, catalyzes the formation of vitamin D_3_ to 25-hydroxyvitamin D_3_ (25(OH)D_3_), the main circulating VITD metabolite, and then 25(OH)D_3_ is converted to 1,25-dihydroxyvitamin D_3_ (1,25[OH]_2_D_3_), the most active form by the enzyme 1 alpha-hydroxylase (encoded by the *CYP27B1* gene). 1,25[OH]_2_D_3_ is inactivated by 24-hydroxylase (encoded by the *CYP24A1* gene) [42]. Megalin and cubilin, endocytic receptors in the cell membrane, allow the internalization of 25(OH)D_3_ and 1,25[OH]_2_D_3_ into the cell [43]. We demonstrated that the ARPE-19 cell line highly expressed *CYP2R1*, *CYP27B*, and *CYP24A* genes and showed a low expression of *CYP27A* and *cubilin* genes; however, they did not express the megalin gene. The HREC cell line highly expressed the *CYP2R1* and *CYP27B* genes. HREC showed a lower expression of *VDR*, *CYP27A*, *cubilin*, and *megalin* genes and did not express the *CYP24A* gene. This is the first time that the machinery for VITD internalization and metabolization is reported in HREC cells. A recent study reported that VITD treatment enhanced VDR expression in ARPE-19 cells treated with H_2_O_2_ [27].

The antioxidant role of vitamin D has been demonstrated in many other micro-environments in the body, especially in the context of diabetes or obesity, including the liver [44], the kidney filtration [45], the heart [46], the hippocampus [47], and the adipose tissue [48], among others. Recently, it has been discovered that vitamin D is not only produced systemically at the renal level, but that extrarenal production is also important. In the eye, there are several tissues in which local production of 1.25(OH)2D3 has been demonstrated, including the sclera, corneal endothelium, ciliary body epithelium, and pigment epithelium, with the corneal endothelium being the eye tissue with the highest conversion rate [49]. We did not find studies focused on the permeability of the retinal blood barrier to VITD; however, it has been demonstrated that the retinal blood barrier has a high permeability to lipophilic substances [50] and also, oral supplemented vitamin D increases the concentration in aqueous humor and tears [49]. To better understand the effect of VITD in the retina, ARPE-19 and HREC cells were subjected to oxidative stress and inflammatory conditions that provoked alterations in tight junctions and also apoptotic signs. Some studies have suggested that VITD can protect against the deleterious effects of reactive oxygen species (ROS), free radicals generated during physiological energy production in the mitochondria [51], therefore improving cell viability in ARPE-19 cells [27] and various tissues [16,52,53,54]. In line with these observations, we demonstrated that H_2_O_2_ and LPS induced the alteration and partial loss of tight junction protein organization (i.e., tortuosity and cytosol localization) in ARPE-19 cells and were restored in oxidative and inflammatory conditions by the addition of VITD to the media. However, results observed in protein expression did not show significant differences in the amount of ZO-1. This phenomenon has been also described by other authors, observing that ZO-1 remains localized in junction despite loss of tight junction protein organization by oxidative stress [55], as observed also in our immunofluorescence images. Inflammatory conditions showed a similar behavior on tight junctions, and their expression was not modified but the organization was altered. VITD addition restored morphological alterations observed in immunofluorescence. The increased early and late apoptosis under oxidative stress and inflammatory conditions was also restored by addition of VITD in ARPE-19 and HREC cells, in concordance with other studies after H_2_O_2_ [27] and after high-glucose-induced oxidative stress and inflammation [17].

A recent study showed that VITD treatment also upregulated the expression of antioxidant genes (*catalase*, *CAT*; *superoxide dismutase SOD1* and *SOD2*; *Glutathione peroxidase GPX2* and *GPX3*) in ARPE-19 cells under similar stress conditions [27]. Accordingly, we found that H_2_O_2_-treated ARPE-19 cells had significantly increased oxidative stress, and VITD exposure counteracted this 8-OHdG production under oxidative-induced conditions. Other authors showed that increased ROS production and lipid peroxidation downregulated expression of antioxidant genes, and decreased activities of SOD and catalase induced in high glucose-treated ARPE-19 cells was counteracted by VITD exposure [17].

In addition to oxidative stress, the levels of proinflammatory proteins such as MCP-1, IL-1β, IL-6, IL-8, and TNF-α influence the development of DR [56,57], and increased aqueous concentration of those molecules in eyes with severe non-proliferative DR suggests that inflammatory changes precede the development of neovascularization [58,59]. Endothelial damage is also linked to increased leukocyte adhesion that is explained by the overexpression of endothelial adhesion molecules such as Intercellular adhesion molecule (ICAM)-1, Vascular cell adhesion molecule (VCAM)-1, Platelet endothelial cell adhesion molecule (PECAM)-1, and P-selectin [59,60]. Vascular endothelial growth factor (VEGF) also alters adherens and tight junctional proteins between the endothelial cells [61,62], favoring the infiltration of leukocytes into the retina. This complex of inflammatory events leads to blood–retinal barrier breakdown and with it some of the vision threatening complications such as macular edema. Directly or indirectly VITD regulate over 200 genes involved in cellular proliferation, differentiation, apoptosis, angiogenesis, and inflammation [63]. VITD have shown to prevent, slow the progression of, ameliorate inflammation markers of, or decrease the severity of many immune-related disorders such diabetes mellitus [64,65]. In our study, all the inflammatory cytokines investigated were upregulated under inflammatory conditions. TNF-α did not result in a statistically significant elevation after LPS induction in HREC cells, probably due to the dispersion observed in control samples. Herein, we observed that the addition of VITD to the media downregulated levels of IL-6, IL-8, MCP-1, IFN-γ, and TNF-α in retinal epithelial cells, as also shown by other authors in ARPE-19 cells [66,67]. These results suggest that VITD can control the broad inflammatory spectrum studied that is present in non-proliferative DR, indicating a clear anti-inflammatory response. In the case of retinal endothelial cells, similar results were observed, with the exception of TNF-α that were unmodified and IL-18 and IL-12p70 that were also downregulated. Elevated levels of IL1-β and IL18 have been demonstrated in streptozotocin (STZ)-induced diabetic rats [68]. Similarly, serum IL-18 levels have also been reported to be elevated in type 1 diabetic patients, half of which had a form of DR [68]. IL-1β, IL-18, and IL-1α have pro-inflammatory actions, and in the case of IL-18, a role in angiogenesis [69]. The inflammasome is an oligomer protein complex that triggers the secretion of IL-1β and IL-18 into the extracellular space [70]. Interestingly, the inflammasome has been particularly related to the neovascular pathology occurring in proliferative DR (PDR) [71,72]. While the major pro-inflammatory cytokines such as IL-6, TNF-α, and IFN-γ could be detected both in non-PDR and in PDR eyes, inflammasome-related cytokine, IL-18, and caspase-1 were particularly increased in the eyes of PDR patients [72]. In our study, IL-18 levels were effectively reduced by VITD in endothelial cells, but not in RPE cells, suggesting a main antiangiogenic role under inflammatory conditions. Moreover, the primary leukocyte populations found in the retina during disease are microglia and macrophages, and it is well known that the activation of the inflammasome is an important mechanism by which these cells cause damage in retinal degenerations. VITD could help to reduce macrophage recruiting by reducing MCP1 levels.

Analyzing the secretome in ARPE-19 cells, researchers found that adding TNF-α to the media regulated different proteins secreted by the RPE, which play a critical role in extracellular matrix remodeling, complement network, and angiogenesis [73]. Thus, VITD supplementation could contribute to reduce those effects. A mixture of IFN-γ, TNF-α, and IL-1β has been shown to decrease the expression of specific genes that play an important role in processes, such as visual cycle, epithelial morphology, melanogenesis, and phagocytosis, in cultured ARPE-19 cells [74]. Therefore, downregulation of those levels by VITD may potentially contribute to restore the RPE dysfunction implicated in retinal diseases, including DR [75,76]. It has been demonstrated that a higher secretion of IL-10 would be a protective factor against the development of proliferative DR (PDR) when proinflammatory cytokines, such as IL-1β, are elevated, as shown in vitreous of PDR patients [77]. In our study, VITD maintained high IL-10 levels, suggesting a possible contribution to an anti-inflammatory environment that must be investigated deeply. Surprisingly, in contrast to other authors [17], IL-1β was not reduced by VITD addition. The bioactive IL12p70 molecule is primarily produced by monocytes, macrophages, dendritic cells, and B-cells. The main functions of IL-12 include the promotion of IFN-γ production from natural killer in cell-mediated immunity [78]. IL-10 is a major inhibitor of IL-12 production by decreasing Nuclear factor κB (NF-κB) and activator protein 1 (AP-1) activation and the association of IL-12p40 promoter with RNA polymerase [78].

Consistent with the anti-inflammation and anti-oxidation role, our results suggested that VITD effectively downregulated in vitro the production of targeted cytokines in DR-related stimuli, suggesting that VITD could block retinal inflammation and oxidative stress associated with DR. Retinal pathologies such as DR are complex diseases with several and different processes involved. We evaluated the effect of vitamin D as an option to restore or to help with the damage provoked by oxidative stress and inflammation. However, different therapies could target multiple steps of oxidative stress for the prevention of this multifactorial blinding complication of diabetes. Santos et al. revised therapies with vitamins and supplements used to treat diabetic retinopathy, and there are various strategies in order to prevent superoxide accumulation, maintain mitochondrial homeostasis, or protect against DNA damage [79]. Other molecules could also be beneficial, even in combination with vitamin D. Although promising, one of the main limitations of this research is the use of an immortalized cell line in which some transcription alterations are produced. Therefore, the results obtained need to be confirmed in primary cells and in experimental in vivo models of retinopathy. Whether these results can be replicated in in vivo models of DR and clinical trials remains to be elucidated.

## 5. Conclusions

In summary, VITD could play a role in the protection of the retina and RPE from oxidative stress, inflammation, and apoptosis through the suppression of pro-inflammatory mediators and by enhancing the antioxidant defense capacity. Taking into consideration all the results we have observed, VITD could be a useful candidate in modulating the chronic low-grade inflammation and oxidative stress responsible for the complications in DR. However, further preclinical in vivo tests and DR patient clinical trials are needed to verify the therapeutic potential of VITD.

## Figures and Tables

**Table 1:** Summary of the main vitamin D (VITD) synthesizing genes in human retinal pigment epithelial cells (ARPE-19) and human retinal endothelial cells (HREC).

	*VDR*	*CYP2R1*	*CYP27A*	*CYP27B*	*CYP24A*	*Cubilin*	*Megalin*	*18S*
ARPE-19	++	+++	++	+++	+++	++	−	+++
HREC	++	+++	++	+++	−	++	++	+++

+++: high expression; ++: medium expression; −: no expression.
